# Evidence for alterations in lipid profiles and biophysical properties of lipid rafts from spinal cord in sporadic amyotrophic lateral sclerosis

**DOI:** 10.1007/s00109-024-02419-7

**Published:** 2024-01-29

**Authors:** Mario Díaz, Noemí Fabelo, M. Virginia Martín, Guido Santos, Isidre Ferrer

**Affiliations:** 1 Department of Physics, Faculty of Sciences, University of La Laguna, Tenerife, Spain; 2 Instituto Universitario de Neurociencias (IUNE), University of La Laguna, Tenerife, Spain; 3 Laboratory of Membrane Physiology and Biophysics, School of Sciences, University of La Laguna, Tenerife, Spain; 4 Centro Oceanográfico de Canarias (COC-IEO), Consejo Superior de Investigaciones Científicas, 38180 Santa Cruz de Tenerife, Spain; 5 Department of Biochemistry, Microbiology, Cellular Biology and Genetics. School of Sciences, University of La Laguna, Tenerife, Spain; 6 University of Barcelona, 08907 Hospitalet de LLobregatBarcelona, Spain

**Keywords:** Amyotrophic lateral sclerosis, Lipid rafts, Neurolipids, Cholesteryl esters, Arachidonic acid, Membrane microviscosity, Domain mobility, Neurodegenerative diseases

## Abstract

**Abstract:**

Amyotrophic lateral sclerosis (ALS) is an age-dependent neurodegenerative disease affecting motor neurons in the spinal cord and brainstem whose etiopathogenesis remains unclear. Recent studies have linked major neurodegenerative diseases with altered function of multimolecular lipid-protein complexes named lipid rafts. In the present study, we have isolated lipid rafts from the anterior horn of the spinal cords of controls and ALS individuals and analysed their lipid composition. We found that ALS affects levels of different fatty acids, lipid classes and related ratios and indexes. The most significant changes affected the contents of n-9/n-7 monounsaturated fatty acids and arachidonic acid, the main n-6 long-chain polyunsaturated fatty acid (LCPUFA), which were higher in ALS lipid rafts. Paralleling these findings, ALS lipid rafts lower saturates-to-unsaturates ratio compared to controls. Further, levels of cholesteryl ester (SE) and anionic-to-zwitterionic phospholipids ratio were augmented in ALS lipid rafts, while sulfatide contents were reduced. Further, regression analyses revealed augmented SE esterification to (mono)unsaturated fatty acids in ALS, but to saturates in controls. Overall, these changes indicate that lipid rafts from ALS spinal cord undergo destabilization of the lipid structure, which might impact their biophysical properties, likely leading to more fluid membranes. Indeed, estimations of membrane microviscosity confirmed less viscous membranes in ALS, as well as more mobile yet smaller lipid rafts compared to surrounding membranes. Overall, these results demonstrate that the changes in ALS lipid rafts are unrelated to oxidative stress, but to anomalies in lipid metabolism and/or lipid raft membrane biogenesis in motor neurons.

**Key messages:**

- The lipid matrix of multimolecular membrane complexes named lipid rafts are altered in human spinal cord in sporadic amyotrophic lateral sclerosis (ALS).
- Lipid rafts from ALS spinal cord contain higher levels of n-6 LCPUFA (but not n-3 LCPUFA), n-7/n-9 monounsaturates and lower saturates-to-unsaturates ratio.
- ALS lipid rafts display increased contents of cholesteryl esters, anomalous anionic-to-zwitterionic phospholipids and phospholipid remodelling and reduced sulphated and total sphingolipid levels, compared to control lipid rafts.
- Destabilization of the lipid structure of lipid raft affects their biophysical properties and leads to more fluid, less viscous membrane microdomains.
- The changes in ALS lipid rafts are unlikely related to increased oxidative stress, but to anomalies in lipid metabolism and/or raft membrane biogenesis in motor neurons.

## Introduction

Amyotrophic lateral sclerosis (ALS) is the most common adult-onset motor neuron disease. ALS is a rapidly progressing neurodegenerative disease characterized by the degeneration of motor neurons, leading to paralysis and eventual death. Multiple pathogenic mechanisms, including lipid dysregulation, have been proposed to contribute to ALS [1–4]. Indeed, dyslipidaemia and lipid dysregulation in the central nervous system and peripheral systems are prevalent in ALS patients and have been reported to be clinically associated with disease severity, functional decline and survival [3–9].

However, while such a link between dysregulation of lipid metabolism and ALS has been often reported, lipidome alterations in neural cell membranes from the brainstem and spinal cord along disease progression remain understudied. Initial evidence for molecular alterations in the lipidome of ALS spinal cord was reported by Cutler and coworkers [10], who found accumulations of cholesterol esters, sphingomyelin and ceramides along with increased oxidative stress-induced death of motor neurons in ALS patients [11]. Targeted mass spectrometry studies from post-mortem ALS subjects have revealed an altered lipid profile of spinal cord grey matter. Such alterations consist of elevated levels of several species of cholesterol esters, and also in a range of sphingolipids including sphingomyelin itself, ceramides and complex glycosphingolipids, such cerebrosides, and acidic glycosphingolipids (mainly gangliosides) [7, 12, 13]. Other reports have also found increased levels of triglycerides and lysophospholipids (particularly lysophosphatidylcholine) [14]. Of note, recent multi-omics study analyses of vulnerable motor neurons have shown increased ceramide levels in the spinal cords but not in ocular motor neurons (which are disease-resistant) derived from ALS patients [15].

Different transgenic models of ALS have been developed based on genetic mutations associated with familiar ALS [1]. The most widely used transgenic model carries the G93A mutation of Zn/Cu superoxide dismutase (SOD1), which develops progressive motor neuron degeneration. Noticeably, lipidomic studies have shown that some of the changes observed in sporadic ALS also occur in the spinal cords of hSOD1-G93A transgenic model [10], as well as in the spinal cords and motor cortex of hSOD1-G93A rats [16]. Indeed, the spinal cords of hSOD1-G93A mouse model show elevated levels of specific ceramides, glucosylceramide, gangliosides and cholesterol esters [10]. Noticeably, these results were also observed in the spinal cord of the transgenic *FUS* mice model of ALS (unrelated to mutations in SOD1, and characterized by an aggressive ALS-like phenotype) [17], indicating a degree of pathological convergence of the different genotypes of familial ALS, at least from the lipidomic perspective [7].

Evidence accumulated over the last decades has demonstrated the involvement of lipid alterations in neural membranes in neurodegenerative diseases. In particular, lipid raft microdomains have been shown to exhibit abnormal lipid profiles, which severely affect the functionality of these signalling platforms, likely by altering their physicochemical properties and the lipid-protein and protein–protein interactions [18–23]. Whether these lipid raft alterations are partly determinant or consequence of the neuropathological process is not known, but changes in some lipid species detected in lipid rafts have been demonstrated to occur at very early stages of the disease [22, 24, 25]. For instance, in the case of Alzheimer’s disease, early lipid raft changes in brain cortical and septal tissues are linked to the amyloidogenic processing of APP and contribute to amyloid plaque formation [25, 26]. Likewise, in Parkinson’s disease, lipid raft changes favour the displacement of monomeric α-synuclein outside lipid rafts and the accumulation of oligomers and high molecular weight forms of α-synuclein in non-rafts, as demonstrated in the human frontal cortex [27–31]. More interestingly, such relationships are also detectable in incidental PD, considered an early stage of PD [28, 31].

In this context, we have aimed to perform an in-depth characterization of the lipid matrix of lipid rafts from the spinal cords of control and ALS human subjects. We have further extended the analyses of key lipid variables to extrapolate the biophysical consequences of lipid alterations in ALS lipid rafts. To the best of our knowledge, this study represents the first detailed description of the lipid structure of lipid rafts from the human spinal cord and the first demonstration of lipid alterations in membrane microdomains in amyotrophic lateral sclerosis.

## Materials and methods

### Subjects

The characteristics of cases and post-mortem delay are shown in Table 1. Samples were obtained from the Institute of Neuropathology brain bank (HUB-ICO-IDIBELL Biobank) under the guidelines of the Spanish legislation (Real Decreto 14/2007) and the approval of the Bellvitge University Hospital local ethics committee (PI-2019/108). The duration of the disease was between 18 and 33 months. Control cases did not show clinical evidence of neurologic and mental disease, and the neuropathological examination revealed no lesions other than those indicated in the table. All ALS cases were sporadic. The genetic study of SOD1 showed no mutations; no expansion of G4C2 hexanucleotide repeats in C9orf72 was identified in any case.

**Table 1:** Summary of cases

**Case number**	**Sex**	**Age**	**PMD**	**Neuropathological diagnosis**	**Other neuropathological alterations**
**Amyotrophic lateral sclerosis (ALS)**					
1	M	70	3	ALS bulbar	NFT II, AGD III
2	M	54	5	ALS spinal	NFT II
3	F	59	14	ALS spinal	NFT II, TDP43 (LATE 2)
4	M	76	13	ALS spinal	NFT III
5	M	65	9	ALS spinal	NFT
**No significative lesions (NSL)**					
5	M	43	6	-	-
6	M	80	5	-	NFT III
7	M	39	3	-	-
8	M	46	15	-	-
9	M	47	5	-	-
10	F	71	6	-	NFT III
11	F	64	5	-	NFT II

*PMD *post-mortem delay, *ALS *amyotrophic lateral sclerosis, *NFT *neurofibrillary tangle pathology, Braak stage, *AGD *argyrophilic grain disease, stage; TDP43 proteinopathy (*LATE *limbic age-related TDP-43 encephalopathy)

### Tissue extraction and lipid raft isolation

Samples of the spinal cord were obtained at post-mortem from four ALS cases and six age-matched controls (NSL group), immediately frozen and stored at − 80 °C until use. The anterior horn of the lumbar and cervical spinal cord was dissected in every case and used for the present study.

Lipid raft fractions were isolated from the human brain, as described in detail elsewhere [26, 32]. Briefly, 0.1 g of brain cortex was homogenized at 4 °C in eight volumes of Tris/ClH isolation buffer containing 1% TritonX-100 and a cocktail of phosphatase and protease inhibitors. The homogenate was centrifuged at 500 × g; the supernatant was then collected and mixed in an orbital rotor for 1 h at 4 °C. The new supernatant was mixed with an equal volume of 80% sucrose in isolation buffer and overlaid with 7.5 ml of a 36% sucrose solution and 2.7 ml of a 15% sucrose solution in ultracentrifuge tubes. Sucrose gradients were centrifuged at 150,000 × g for 18 h at 4 °C using a Beckman SW41Ti rotor. The purity of lipid rafts (corresponding to fractions 1 + 2) was checked by using anti-flotillin-1 and anti-SOD1 antibodies in Western blot assays, as described in our previous studies [28, 32].

### Lipid analyses

The lipid composition of spinal cord fractions was analysed according to the method previously reported for isolated lipid rafts [26, 28, 32]. Briefly, total lipids were extracted with chloroform/methanol (2:1 v/v) containing butylated hydroxytoluene (0.01%). Fatty acids were extracted after acid-catalysed transmethylation, and the resultant FAMEs (fatty acid methyl esters) and DMAs (dimethylacetals) were purified by thin-layer chromatography (TLC) and quantified by gas chromatography using a TRACE GC Ultra (Thermo Fisher Scientific). Individual FAME and DMA were identified by reference to a multi-standard mixture (Supelco PARK) and confirmed using a DSQ II mass spectrometer (Thermo Fisher Scientific). Lipid classes were separated by one-dimensional double development high-performance thin-layer chromatography (HPTLC) and were quantified by scanning densitometry using a Shimadzu CS-9001PC dual-wavelength spot scanner.

### Microviscosity analyses

Lipid raft microviscosity was determined following the procedures previously described for human brain cortex raft membranes [24, 33]. Apparent microviscosities (*η*_app_) were estimated at the membrane plane from TMA-DPH steady-state anisotropies using Perrin equation-based regression analyses for *η*_app_ and lipid variables [25, 34].

### Mathematical modelling

We used an agent-based mathematical model to predict the 2D mobility of the lipid elements in the cell membrane, the lipid raft composition and their physical properties. The model was initially designed to explain changes in frontal cortex lipid rafts in WT and APP/PS1 transgenic mice [21] and subsequently optimized to predict lipid raft changes in human brains [23]. Seven lipid groups were included in the model, i.e., cholesterol (CHO), sterol esters (SE), docosahexaenoic acid (DHA), n-6 long-chain polyunsaturated fatty acids (LCPUFA), monounsaturated fatty acids (MUFA), saturated fatty acids (SFA) and sphingolipids.

### Statistical methods

All lipid variables were initially assessed by non-parametric Mann–Whitney *U* test for differences between control and ALS groups and ANOVA-like Cohen’s *d* for quantification of effect sizes [35, 36]. Individual lipid classes and fatty acids were submitted to multivariate analyses using principal components analysis (PCA), in order to obtain the extraction coefficient vectors and contributions to overall variance. Factor scores were further analysed by one-way ANOVA to assess the differences in lipid signatures. Pearson’s correlation, simple and multiple linear regression, ANCOVA and Cohen’s *f*^2^/R^2^ analyses were performed to assess the statistical significances and effect sizes of bivariate and multiple relationships between different lipids and groups.

## Results and discussion

### Alterations in levels of fatty acids and lipid classes in lipid rafts from ALS samples

**Fig. 1:**
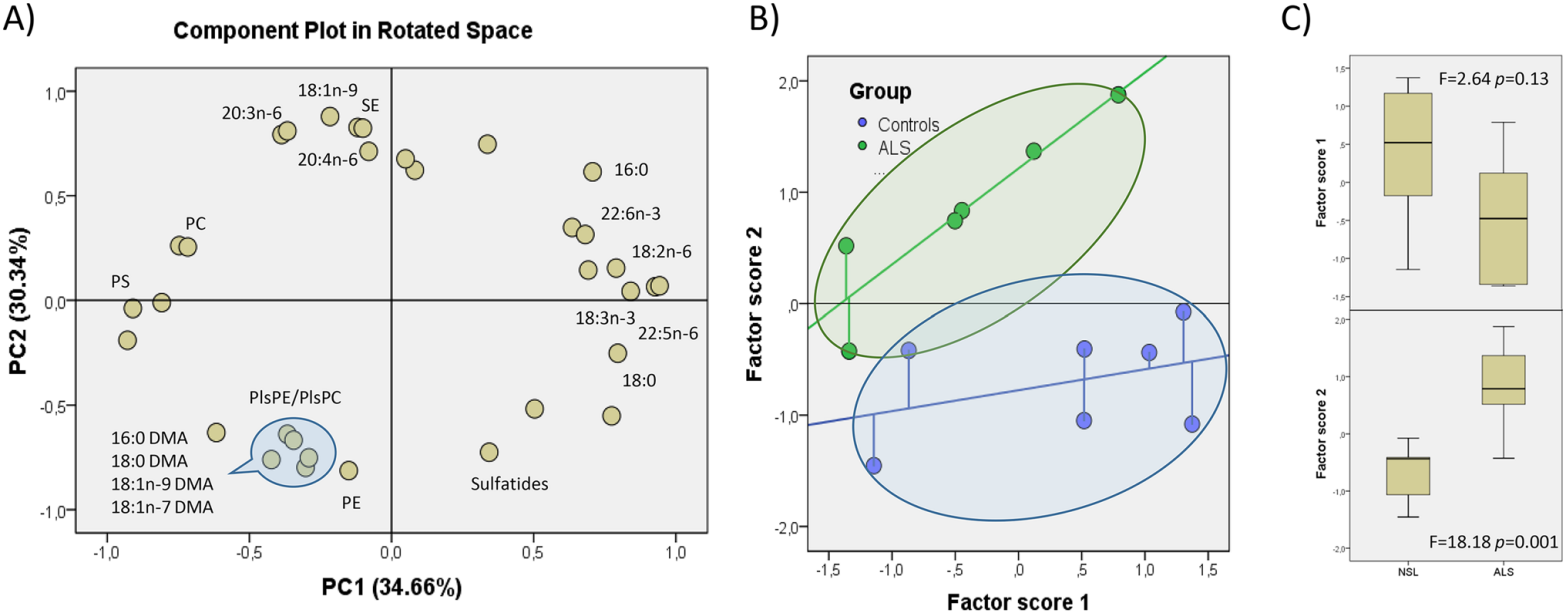
Multivariate analyses of lipid profiles in lipid rafts from spinal cords of control and ALS subjects. **A** Scatter plot of principal components (PC1 and PC2) for individual lipid species in rotated space. Values in parentheses indicate the percent of variance explained by each principal component. DMA, dimethylacetals; PE, phosphatidylethanolamine; PlsPE/PlsPC, phosphatidylethanolamine/phosphatidylcholine plasmalogens. **B** Scatter plots for factor scores from individuals from control and ALS groups. **C** Box plots and ANOVA results for factor scores 1 and 2

In order to analyse the lipid profiles of lipid rafts from ALS and NSL groups, we designed a whole database including new variables for indexes, ratios and totals relevant to lipid raft biochemical structure. Lipid data was initially submitted to PCA and the results are shown in Fig. 1. The two principal components explain 68% total variance, with similar contributions (above 30% each) for components 1 and 2. PC1 was positively related to saturates (16:0 and 18:0), n-6 PUFA (18:2n-6 and 22:5n-6) and n-3 PUFA (18:3n-3 and DHA) and negatively to lipid classes phosphatidylcholine (PC) and phosphatidylserine (PS). PC2 was associated with n-6 LCPUFA (20:4n-6 and 20:3n-6), monoenes (18:1n-9 and 18:1n-7) and cholesteryl esters (SE) and negatively to phosphatidylethanolamine (PE), sulfatides and dimethylacetals (16:0 DMA, 18:0 DMA, 18:1n-9 DMA and 18:1n-7 DMA) which are indirect indications of plasmalogens. The scatterplot of factor scores for each principal component discloses two groups of lipid rafts with a high degree of segregation (Fig. 1B). One-way ANOVA revealed differences between groups within factor scores, which were statistically significant for factor score 2 (Fig. 1C). Interestingly, factor scores exhibited linear relationships (*R*^2^ = 0.847 in ALS vs *R*^2^ = 0.149 in NSL) with regression coefficients significantly larger for ALS group (*p* < 0.05).

**Fig. 2:**
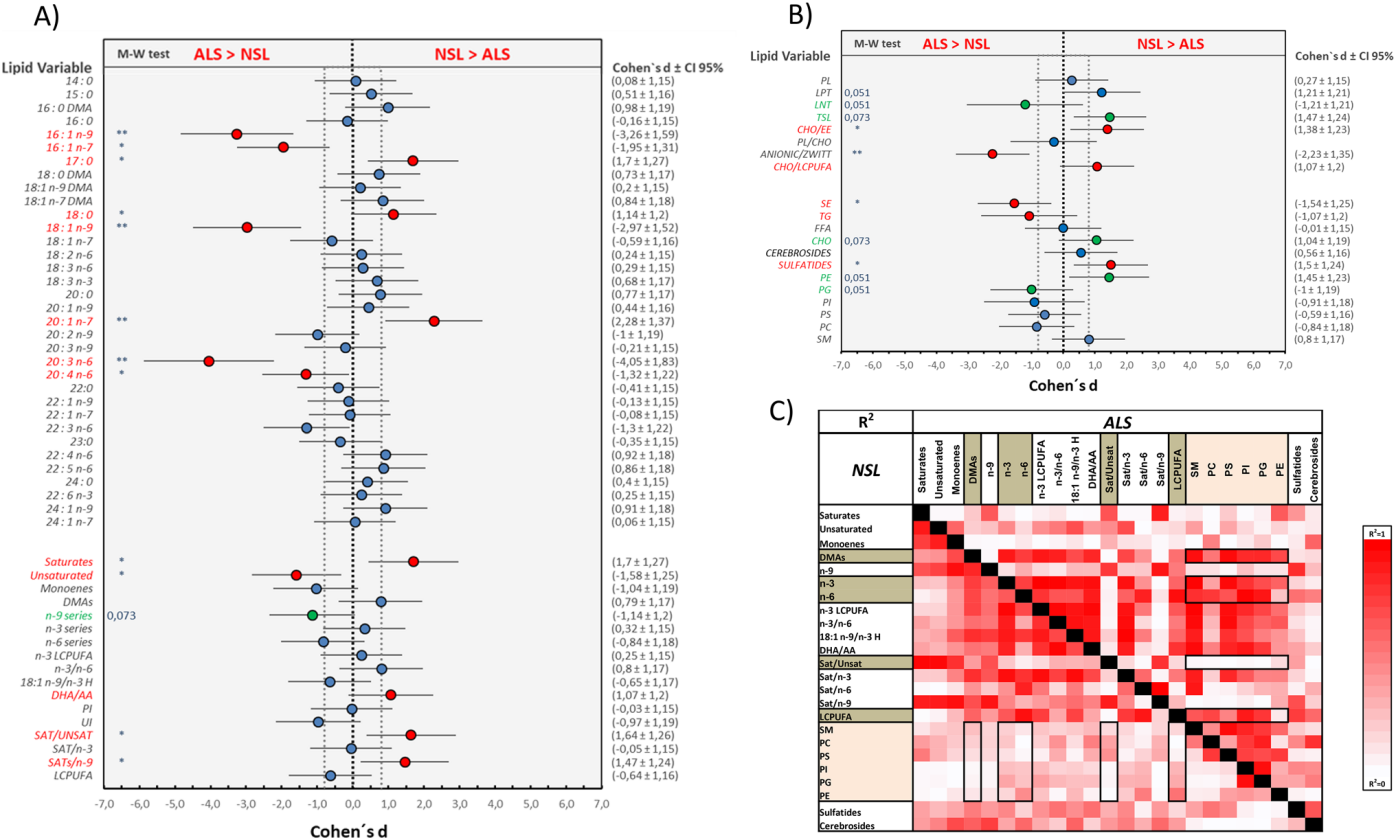
Lipid profiles in lipid rafts from control (NSL) and ALS spinal cords. **A** Forest plot for individual fatty acids, totals and indexes. **B** Forest plot for individual lipid classes, totals and indexes. Data in **A** and **B** are represented as Cohen’s *d* value ± 95% CI to indicate effect sizes. M-W test—*p*-values from Mann–Whitney *U* test. Dashed lines around zero indicate the threshold for large effects (± 0.8). *p < 0.05, **p < 0.01. **C** Heatmap representation of correlation effect sizes (*R*^2^) for the relationships between main fatty acid variables and phospholipids/sphingolipids groups in lipid rafts from ALS and control (NSL) groups. Black rectangles indicate the main significant differences according to Cohen’s *f*

Detailed analyses of fatty acids (Fig. 2A) indicate that lipid rafts from ALS group contain larger levels of n-9 and n-7 monoenes (16:1n-9, 18:1n-9, 16:1n-7 and 20:1n-7) and lower saturates (especially stearic acid, 18:0). The remarkable increase in n-9 monoenes 18:1n-9 and 16:1n-7 (oleic and palmitoleic acids, respectively) likely reflects the increased activity of stearoyl-CoA desaturase (SCD-1 or Δ-9 desaturase) in ALS neurons. It is worth mentioning the significant change observed for hypogeic acid (16:1n-9), which was 85.6% higher in ALS samples. As this fatty acid is a beta-oxidation product of 18:1n-9 (and not produced by Δ-9 desaturase), it suggests an alteration in mitochondrial beta-oxydation, which might influence the overall oxidative conditions in spinal motor neurons.

Consequently, lipid rafts from ALS spinal tissue contain higher unsaturated and lower saturate levels, which is reflected in the significant reduction of the saturates-to-unsaturates ratio (Sat/Unsat). No changes were observed for fatty acids of the n-3 series, including DHA, which underwent only a slight reduction (− 6.5%). However, some of the LCPUFA from the n-6 series were significantly augmented in ALS; in particular, for arachidonic acid (AA, 20:4n-6) and 20:3n-6, increases were + 16.5% and + 68.8%, respectively. These opposed changes in LCPUFA were responsible for the small effect of the disease on the peroxydability and unsaturation indexes (PIx and UIx, respectively) of lipid rafts. The elevated arachidonic acid has been proposed to play a role in the selective vulnerability of spinal cord motor neurons in ALS [7, 15]. Of note, a recent study in ALS patients has identified a discriminatory subset of plasma metabolites, which include elevated levels of arachidonic acid, that correlates positively and with high specificity with disease severity [37]. A possible association between increased arachidonic acid levels and ALS might be linked to the hydrolysis of membrane phospholipids by cytosolic phospholipase A2 (cPLA2), which would give rise to proinflammatory eicosanoids contributing to neurotoxicity via activation of neuroinflammation. In support of this, the expression and activity of cytosolic PLA2 have been found to be increased in the spinal cords of ALS patients and also in motor neurons from hSOD1-G93A mice [38, 39].

Relevant changes in lipid classes were also observed in ALS spinal cord (Fig. 2B). The most significant changes were the 223% increase in cholesteryl esters (SE) and the 17.9% reduction of sulfatides (sulphated sphingolipids, *p* < 0.05, Cohen’s *d* = 1.503), which mostly accounts for the decrease in total sphingolipids (TSL, *p* = 0.073, Cohen’s *d* = 1.47). ALS lipid rafts also displayed alteration in glycerophospholipids, consisting in the reduction of PE (− 12.9%) and increased levels of glycerophosphoglycerol (PG) (+ 69.2%), glycerophosphoinositol (PI) (+ 22.5%) and PS (+ 17.46).

In agreement with our findings, a recent study using multi-omics approaches in iPSC-derived neurons from ALS patients has revealed higher levels of arachidonic acids and certain phospholipids (PE, PS, PG and lysophospholipids) compared to control motor neuron cultures [15]. Interestingly, these effects were specific for spinal cord motor neuron cultures and not observed in ocular motor neuron cultures derived from human ALS-iPSCs cells [15].

### Evidence for disease-related phospholipid remodelling

The first indication for the occurrence of phospholipid remodelling was the significantly higher anionic-to-zwitterionic phospholipids ratio (*p* < 0.01, Cohen’s *d* =  − 2.23) in ALS lipid rafts (Fig. 2B), which due to both reduction of neutral PE and increased levels of all anionic phospholipids, i.e. PG, PI and PS.

Therefore, we next performed analyses of effect size using the determination coefficient (*R*^2^) to assert the fractional variance shared by each pair of variables. The results shown in Fig. 2C revealed a significant overall difference between ALS and NSL groups, with the former showing higher degrees of bivariate associations compared to NSL, particularly for glycerophospholipids and specific fatty acid groups and indexes (highlighted in rectangles in Fig. 2C). Strongest size effects were observed for total DMAs, total n-3 (mostly represented by DHA), total n-6 (mostly represented by arachidonic acid), total LCPUFA and Sat/Unsat, yet the magnitude depended on the type of phospholipid. For instance, the Sat/Unsat ratio for PC class indicates a strong covariation in NSL compared to ALS (*R*^2^ values 0.36 vs 0.01, respectively), very similar to PS, where *R*^2^ values for the same index were 0.43 (NSL) and 0.00 (ALS). Effect sizes were severely altered for total LCPUFA, total n-3, total n-6 and total DMAs in all phospholipids, with *R*^2^ values being considerably larger in the ALS group compared to NSL. These results pinpoint a strong remodelling of lipid raft phospholipids by the effect of ALS.

The relationships between individual phospholipids and individual fatty acids were further assessed by Pearson’s correlation (Table 2). The results demonstrated important changes in the association of glycerophospholipids and fatty acids, often exhibiting opposed relationships between the two groups. Thus, the relevant positive relationships between total PE and DHA (*r* = 0.48, *p* < 0.05) and AA (*r* = 0.72, *p* < 0.01) observed in NSL were inverted in ALS (DHA *r* =  − 0.75, *p* < 0.01; AA *r* =  − 0.94, *p* < 0.01). Unlike PE, PC correlations with DHA and AA were either negative (DHA *r* =  − 0.42, *p* < 0.05) or absent (AA *r* = 0.12, *p* > 0.05) in NSL but remained similar in ALS. Within anionic phospholipids, PS was the only PL which displayed disease-related changes, being poorly related to AA and DHA in NSL (*r* = 0.31, *p* > 0.05 for AA and *r* =  − 0.23, *p* > 0.05 for DHA) but strongly negative in ALS (*r* =  − 0.81, *p* < 0.01 and *r* =  − 0.97, *p* < 0.01 for AA and DHA, respectively). Regarding DMAs, as surrogates of plasmalogens, PE was either negatively or poorly related in NSL, but strongly, and positively, correlated in ALS (*r* = 0.84, *p* < 0.01 and *r* = 0.87, *p* < 0.01, for 16:0 DMA and 18:1n-9 DMA). On the contrary, positive relationships were observed for PC and 16:0 DMA (*r* = 0.37 *p* < 0.05), 18:1n-9 DMA (*r* = 0.60, *p* < 0.05) and 18:1n-7 (*r* = 0.67, *p* < 0.05) in NSL, which were retained (even enhanced) in ALS. These observations indicate a selective remodelling of PE plasmalogens during the development of amyotrophic sclerosis. Recall that the predominant ether lipid form in nerve cell membranes derives from glycerophosphoethanolamine and corresponds to 1-(1Z-alkenyl),2-acylglycerophosphoethanolamines (PlsEtn, PE plasmalogen or PE(P-)), accounting for 50–60% of the total PE class [40–42]. On the other hand, anionic phospholipids also displayed disease-related changes. The main anionic phospholipid, PS, was strongly correlated to 16:0 DMA (*r* = 0.98, *p* < 0.01) in ALS but not in NSL (*r* = 0.25, *p* > 0.05), while AA was positively associated with PS in NSL (*r* = 0.31, *p* < 0.05) but negatively in ALS (*r* = 0.81, *p* < 0.01).

**Table 2:**
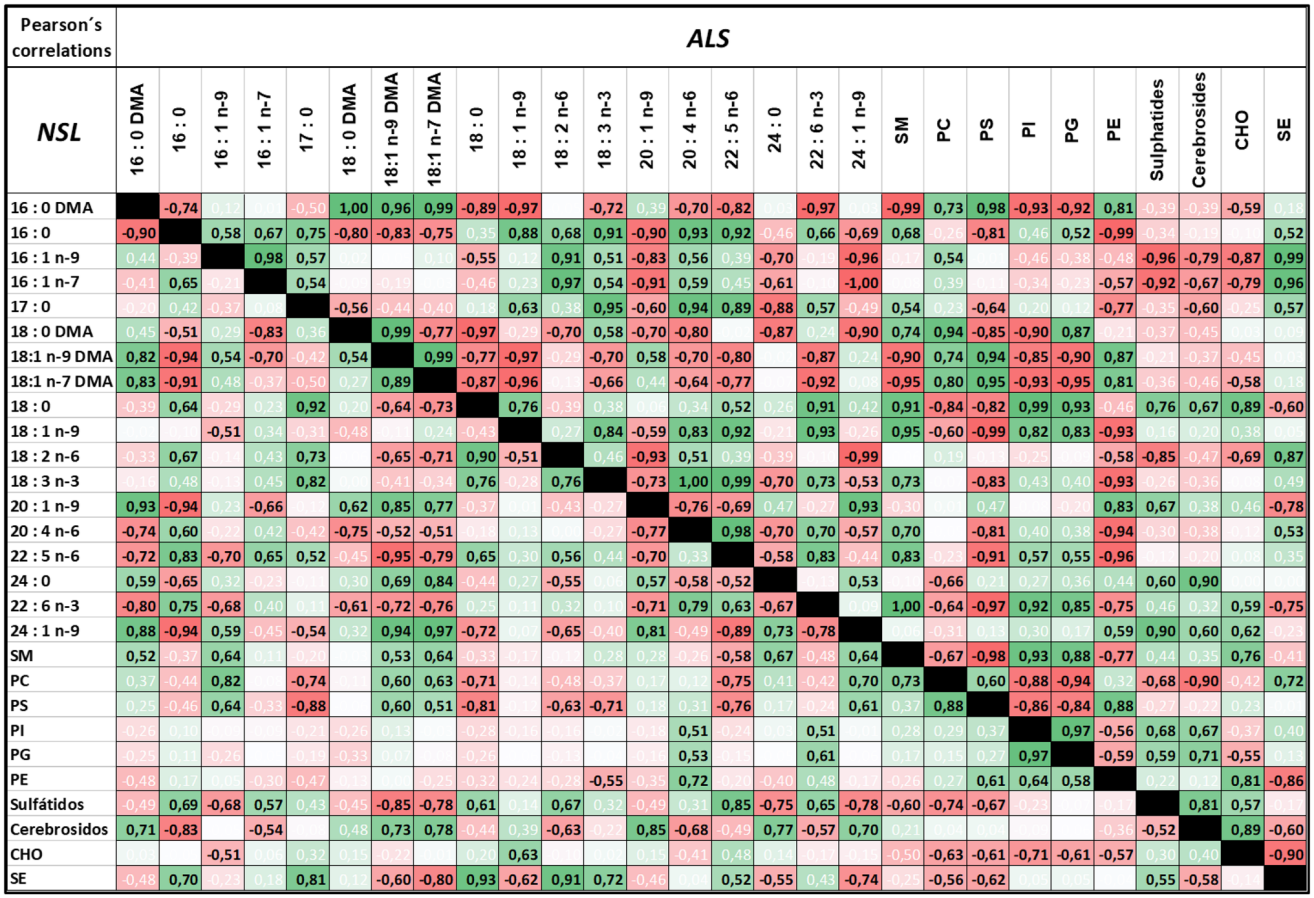
Pearson’s correlation analyses for main fatty acids and lipid classes in NSL and ALS groups

Significant correlation values (*p *> 0.05) are highlighted in bold

In summary, we conclude that besides differences in the anionic-to-zwitterionic proportions, the development of the disease alters the contents and compositional structure of glycerophospholipids and their derived plasmalogens.

### Changes in the lipid raft cholesterol-sterol(cholesteryl) ester binary system

**Fig. 3:**
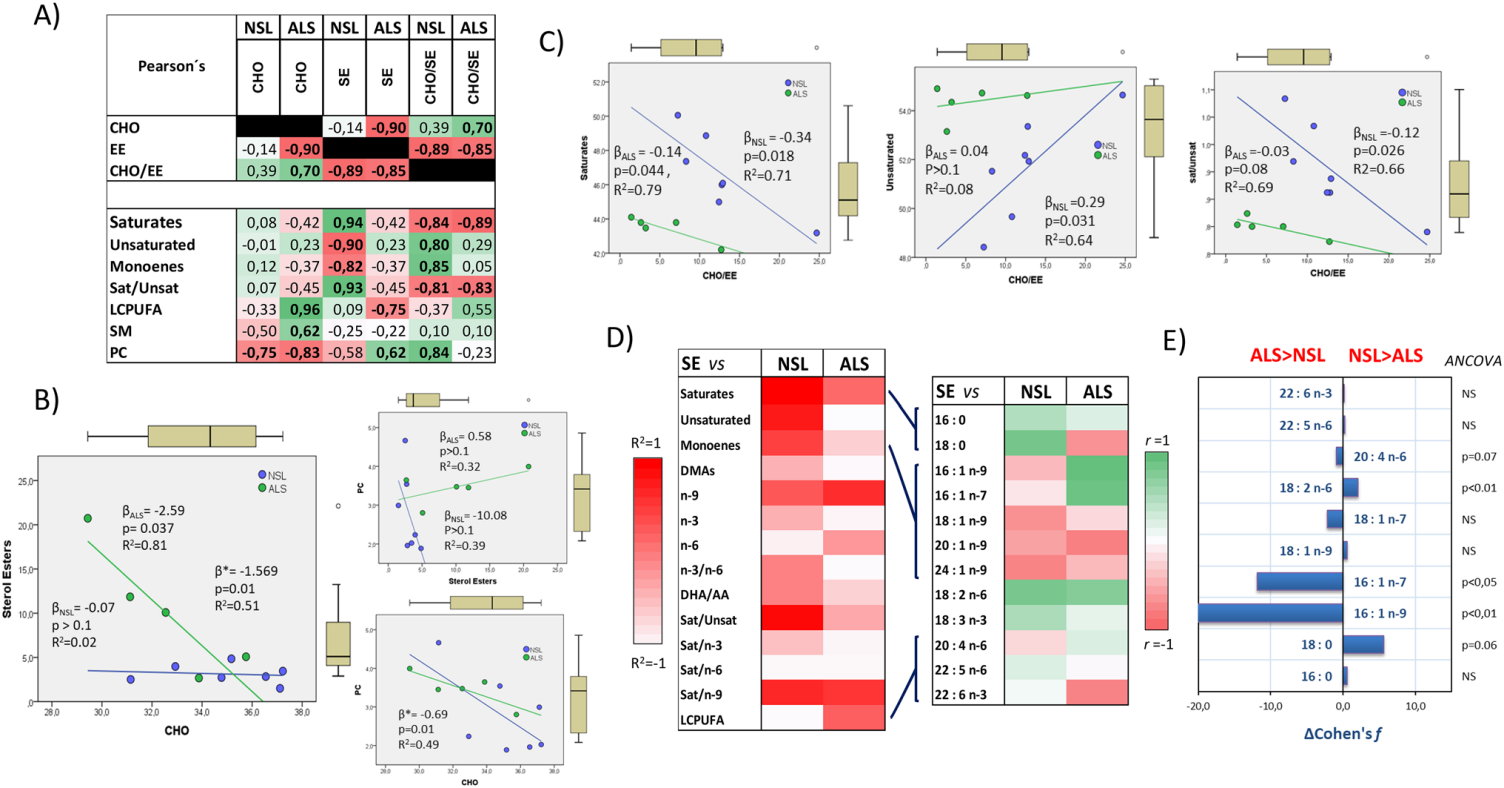
Cholesterol-sterol(cholesteryl) ester relationships in NSL and ALS lipid rafts. **A** Pearson’s correlations between cholesterol (CHO), cholesteryl esters (SE), CHO/SE ratio, fatty acid groups, saturates-to-unsaturates (Sat/Unsat) ratio, sphingomyelin (SM) and phosphatidylcholine (PC) in lipid rafts. Bold numbers indicate significant correlation values. **B** Regression analyses of CHO, sterol esters and PC in NSL and ALS lipid rafts. Box plots for each dependent and independent variable are shown in the plots. Β, regression coefficient; *R*^2^, determination coefficient. **C** Regression analyses CHO/SE ratios as independent variables and saturates, unsaturates and Sat/Unsat ratios as dependent variables in NSL and ALS samples. Box plots for each dependent and independent variable are shown in the plots. **D** Heatmap representation for correlation effect sizes (*R*^2^) for the relationships between SE and main fatty acid variables (left panel). Correlation coefficients for SE and specific fatty acids are illustrated in the right panel. **E** Effect size analyses (Cohen’s *f*) and covariate analyses (ANCOVA) for regression coefficients of relationships between SE and fatty acids analysed in **D**

We showed above the significantly higher levels of sterol esters and moderate-to-strong reduction of cholesterol in ALS lipid rafts. Bivariate analyses revealed that such changes are significantly correlated in ALS but not in NSL (Fig. 3A). Regression analyses revealed a negative linear relationship between CHO (independent variable) and SE (dependent variable) in the whole dataset (Fig. 3B) with slope (β*) of − 1.57. Moreover, inter-group analyses indicated that most of such negative associations derived from ALS lipid rafts (*β*_ALS_ =  − 2–59 compared to *β*_NSL_ =  − 0.07, *F* = 15.55, *p* = 0.004), which indicates that esterification of cholesterol is strongly favoured in ALS. 103The esterification reaction of cholesterol in nerve cells is mostly catalysed by acyl coenzyme-A cholesterol acyltransferase (ACAT1) in the endoplasmic reticulum [43]. Alternatively, cholesterol esters may be synthesized by lecithin:cholesterol acyl transferase (LCAT), with concomitant formation of 1‑acyl-lysophosphatidylcholine (LPC) [43]. However, this second route may be discharged in ALS lipid rafts because of (1) the levels of LPC that were undetectable in either group (Fig. 2A), (2) the absence of significant associations between PC and sterol esters (Fig. 3B) and (3) the negative relationship between PC vs cholesterol, which was unaffected by the disease (Fig. 3B). Therefore, the cholesterol-to-sterol ester ratio (CHO/SE) may be used as an indicator of ACAT1 activity. Hence, as CHO/SE was significantly reduced in ALS lipid rafts by 57.6%, it may be accepted that the enormous increase in SE levels (ALS > NSL) derives from increased ACAT1 activity and/or expression in ALS neurons. In agreement with our findings, several studies in ALS models and human spinal cord have reported increased levels of cholesteryl esters in whole grey matter preparations (10, 14, 16). Further, increased levels of ACAT1 and cPLA2 mRNA have been demonstrated in the hSOD1-G93A transgenic ALS model [14]. In human grey matter, these changes correlated with increased activity of cPLA2 [14, 38], the enzyme responsible for supplying fatty acids to ACAT1 to generate SE upon activation by esterification with CoA [43].

Besides total levels of cholesteryl esters, lipidomic studies have demonstrated selective ALS-related alterations in the fatty acid composition of SE molecular species, in particular concerning saturates, monounsaturated and polyunsaturated fatty acids, in the spinal cord [14, 16]. Mass spectrometry-based techniques were not suitable for the lipid raft preparations in the present study; therefore, we used a statistical approach to assess the potential relationships between specific fatty acids and SE in lipid rafts. We initially considered fatty acids that were significantly correlated to SE in Table 2 and/or exhibited strong effect sizes in Fig. 2C in either group. Effect sizes for correlation analyses for SE on fatty acid groups and indexes are shown in Fig. 3D (left panel). Major differences between NSL and ALS groups were observed for total unsaturates, total LCPUFA, n-6 series and Sat/Unsat and n3/n6 ratios. Cholesteryl esters were similarly related to long-chain n-9 fatty acids (18:1n-9, 20:1n-9 and 24:1n-9) as well as to 18:2n-6 in both groups (Fig. 3D, middle panel). However, correlation analyses of SE on individual fatty acids revealed opposed relationships for specific fatty acids from all groups, i.e. saturates (18:0), unsaturates (16:1n-7 and 16:1n-9) and LCPUFA (22:6n-3) (Fig. 3D, middle panel). Covariance analyses and Cohen’s *f* on regression coefficients shown in Fig. 3E indicate higher esterification rates for 16C monoenes (16:1n-7 and 16:1n-9) and lower for 18:0, 18:1n-9 and 18:2n-6 in ALS lipid rafts, compared to NSL counterparts. Although using a different methodology, our results substantially agree with the observations reported recently in whole lipids from spinal motor neurons in ALS subjects [14] and mutant hSOD1-G93A mice [14, 16]. Noticeably, these studies also showed increased esterification of SE with arachidonic acid as a signature of ALS, a finding that was also observed here (Fig. 3D) in spinal cord lipid rafts (*p* = 0.072, Cohen’s* f* =  − 0.88). Therefore, we may conclude that lipid rafts undergo changes in cholesteryl ester species, which, at least in part, result from altered ACAT1 activity in ALS motor neurons.

Recent studies in ALS have revealed disturbances in the mitochondria-associated membrane systems called MAMs [44, 45]. MAMs are specialized endoplasmic reticulum (ER)-like membrane subdomains tightly associated with mitochondria, which are endowed with physical and biochemical properties of lipid rafts [46, 47]. There exist essential links between our present results and MAM-associated enzymes for lipid biogenesis. First, we detected lower levels of phosphatidylethanolamine and higher anionic/zwitterionic ratios in ALS samples, which is coherent with augmented PSS1/2 (phosphatidylserine synthase-1/2) activities. Second, the high abundance of cholesteryl esters and lower CHO/SE in ALS are mediated by increased ACAT1 activity and/or expression, and third, the larger amounts of AA in ALS phospholipids are most likely due to increased ACSL4 (long-chain fatty acid-CoA ligase type 4) activity, the enzyme determining the rate of arachidonoyl-CoA synthesis and their incorporation into glycerophospholipids. Further studies on MAMs are necessary to elucidate whether the altered lipid profiles observed in lipid rafts are derived from altered MAMs.

### Evidence for alterations in lipid raft microviscosity and membrane domain fluidity and mobility

**Fig. 4:**
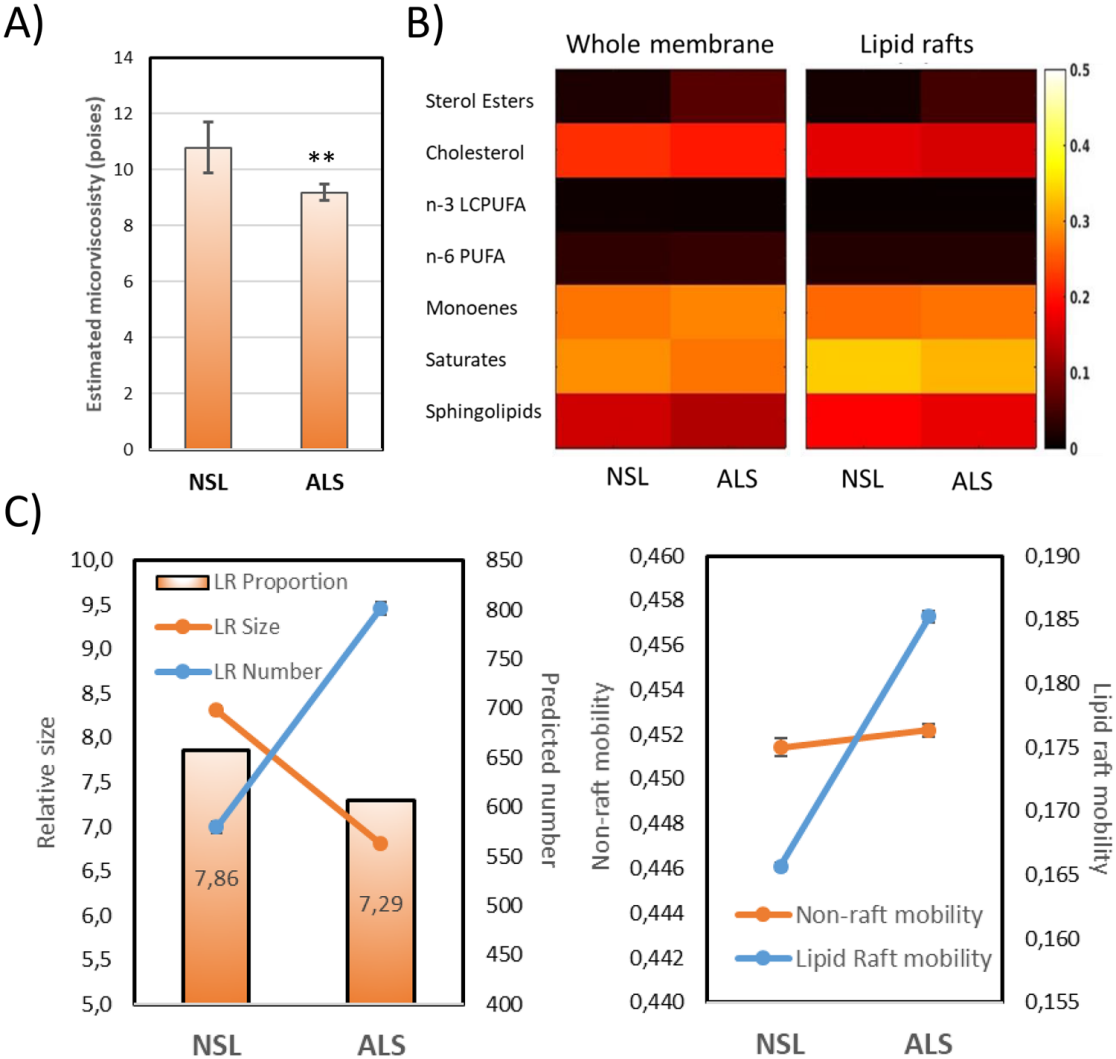
Biophysical and dimensional correlates of lipid profiles in lipid rafts from NSL and ALS groups. **A** Estimated microviscosities of NSL and ALS lipid rafts as assessed at the membrane plane. **p < 0.01. **B**, **C** Results from mathematical agent-based simulations. **B** Heatmap of simulated group lipid contents in whole membranes and lipid rafts in NSL and ALS groups. **C** Left panel—model predictions for lipid raft sizes, number and membrane proportions. Right panel—predictions for lipid rafts and non-raft mobility

In order to estimate potential changes in lipid raft biophysical properties as a result of differences in lipid profiles between NSL and ALS preparations, we used the multiple regression models approach obtained for lipid rafts from human and mice nerve cell membranes [25, 34]. We observed a significant reduction of lipid raft microviscosity in ALS as compared to NSL groups (Cohen’s *d* = 2.71, *p* < 0.01), representing a 14.8% increase in membrane fluidity in ALS membranes (Fig. 4A). This observation agrees with the changes in the values of n-6 LCPUFA, 18:1n-9 and Sat/Unsat observed in ALS, which collectively point to more fluid membranes in the diseased motor neurons. So far, only one study has evaluated membrane fluidity in ALS nerve cells [48]. By measuring steady-state TMA-DPH anisotropy in the transgenic SOD1G93A mice, the authors report reduced membrane fluidity in the spinal cord compared to wild-type [48]. The authors explained this result as a consequence of increased lipid peroxidation of PUFA in membrane phospholipids in response to deficient SOD1 activity [48]. However, the hypothesis that membrane destabilization in ALS is secondary to lipid peroxidation initiated by mutated SOD1-induced ROS and oxidative stress is unlikely for two reasons. First, LCPUFA levels (in particular n-6) were not decreased in ALS lipid rafts, and second, most ALS subjects do not carry mutated *SOD1* genes (approximately 20% of familial ALS, or < 0.2% of total cases) [2, 11]. Instead, according to our data, changes in membrane fluidity occur in the opposite direction and in response to dysregulation of lipid metabolism and/or membrane biogenesis.

We also used an agent-based mathematical model [21, 23] to estimate both fluidity and dimensional changes in ALS lipid rafts. Based on the lipid composition (Fig. 4B), the agent-based model predicted the increase in lipid raft mobility in lipid rafts but not in non-raft domains (Fig. 4C), which agrees with the estimations of membrane microviscosity, and suggests the selective alteration in lipid rafts. In addition, model output indicates a reduction in the membrane proportion of lipid raft, as well as smaller sizes (Fig. 4D), which would commit the spatio-temporal dynamics of lipid rafts. In the predicted scenario, the membrane of ALS neurons contains more mobile small-sized lipid rafts, which are endogenously more fluid than in non-diseased motor neurons. Clearly, these results are relevant from the pathophysiological perspective since they suggest a more loosely packing between membrane lipids and lower restrictions for protein lateral displacements. This likely affects protein clustering and signalling processes. In agreement with this, proteomic analyses of membrane lipid rafts in SOD1-G63A spinal cords have demonstrated a differential expression pattern of an important number of raft-associated proteins involved in neurotransmitter synthesis and release, cytoskeleton organization, vesicular transport and linkage to the plasma membrane [49]. Also, there is evidence in transgenic mice and human ALS that impaired neurotrophic signalling associates with ALS [50, 51], and that deficient neurotrophic signalling associates with increased neuronal susceptibility to excitotoxicity, which is proposed as an incipient mechanism underlying motor neuron vulnerability in ALS [51, 52].

## Conclusions

We propose here that the destabilization of the membrane lipid composition of lipid rafts along with the consequences in their biophysical properties is the key factor underlying cellular anomalies in motor neuron physiology during the development of sporadic amyotrophic lateral sclerosis. Such membrane alterations might help to explain the connexions between different pathological events all conveying to the selective vulnerability of motor neurons, such as glutamatergic excitotoxicity, oxidative stress, neuroinflammatory response and probably protein aggregation through membrane interactions, as reported in other proteinopathies.

The present lipid alterations were observed in sporadic ALS. Additional studies on familial ALS linked to SOD1 mutations and sporadic and familial cases linked to C9orf72 expansions will increase our knowledge of commonalities and differences in lipid raft composition within the ALS spectrum. Nevertheless, we reckon this initial study is relevant as it highlights the potential involvement of key membrane mechanisms during the early development of amyotrophic lateral sclerosis.

## Data Availability

The datasets generated during and/or analysed during the current study are available from the corresponding author on reasonable request.
